# Transcriptome Analysis Identifies Key Genes Involved in Response and Recovery to High Heat Stress Induced by Fire in *Schima superba*

**DOI:** 10.3390/genes15081108

**Published:** 2024-08-22

**Authors:** Shujing Wei, Yingxia Zhong, Wen Wen, Chong Yu, Ruisen Lu, Sisheng Luo

**Affiliations:** 1 Guangdong Academy of Forestry, Guangzhou 510520, China; weishujing2003@163.com (S.W.);; 2 Guangzhou Institute of Environmental Protection Science, Guangzhou 510520, China; wenwen08212024@163.com; 3 Jiangsu Key Laboratory for the Research and Utilization of Plant Resources, Institute of Botany, Jiangsu Province and Chinese Academy of Sciences, Nanjing 210014, China

**Keywords:** *Schima superba*, fire-resistant tree species, heat stress, transcriptome, differentially expressed genes

## Abstract

Fire-resistant tree species play a crucial role in forest fire prevention, utilizing several physiological and molecular mechanisms to respond to extreme heat stress. Many transcription factors (TFs) and genes are known to be involved in the regulatory network of heat stress response in plants. However, their roles in response to high temperatures induced by fire remain less understood. In this study, we investigated *Schima superba*, a fire-resistant tree, to elucidate these mechanisms. Leaves of *S. superba* seedlings were exposed to fire stimulation for 10 s, 30 s, and 1 min, followed by a 24-h recovery period. Fifteen transcriptomes were assembled to identify key molecular and biological pathways affected by high temperatures. Differentially expressed genes (DEGs) analysis revealed essential candidate genes and TFs involved in the heat stress response, including members of the ethylene-responsive factors, WRKY, MYB, bHLH, and Nin-like families. Genes related to heat shock proteins/factors, lipid metabolism, antioxidant enzymes, dehydration responses, and hormone signal transduction were differentially expressed after heat stress and recovery, underscoring their roles in cellular process and recovery after heat stress. This study advances our understanding of plant response and defense strategies against extreme abiotic stresses.

## 1. Introduction

Global climate change has led to an increase in the frequency and intensity of forest fires, posing significant threats to ecosystems and human safety [1]. The occurrence of wildfires not only results in the loss of biodiversity and land degradation but also has adverse effects on atmospheric environments [2]. Fire-resistant tree species are commonly used as fire isolation belts and play a crucial role in mitigating the impact of wildfires on ecosystems and human communities. These species have developed mechanisms to endure and recover from the intense heat associated with fire-prone environments [3,4]. Understanding the physiological and molecular basis of heat stress induced by high temperature in fire-resistant trees is essential for developing effective fire management strategies and selecting appropriate species for reforestation in fire-prone areas.

Fire-resistant tree species have a range of physiological and molecular adaptations that enable them to survive and recover from heat stress induced by high temperature [5]. Thick bark acts as a heat shield, insulating the underlying tissues from extreme temperatures and preventing damage to the cambium layer. Additionally, some species have evolved strategies to regulate leaf temperature through transpiration and leaf orientation, reducing the risk of overheating and dehydration during fires [6,7]. Other adaptations include the accumulation of heat shock proteins (HSPs) and antioxidants, which play crucial roles in protecting cellular structures and maintaining homeostasis under heat stress conditions [8,9]. On the molecular level, these species activate a suite of genes involved in heat stress response pathways, including those coding for HSPs, which are regulated by heat stress transcription factors (HSFs) [10,11]. Transcriptomic analyses have shown that genes encoding HSPs, chaperones, and antioxidant enzymes are up-regulated in response to heat stress [12,13]. A variety of transcription factors (TFs) and signaling molecules regulate the expression of these heat stress response genes [14,15], providing valuable insights into the molecular mechanisms underlying heat tolerance in fire-resistant trees.

*Schima superba* Gardner & Champ, an evergreen broad-leaved tree in the *Theaceae* family, is commercially valued for its timber and fire protection [16,17]. Known for its strong fire resistance, *S. superba* is widely used as a fire break in tropical and subtropical regions in China. Previous studies have explored its leaf structural characteristics in capturing smoke particles following forest fire [18]. In the aspect of heat release rate (HRR) and total heat released (THR), *S. superba* has demonstrated superior performance, making it more suitable for use as a fuel break in southern China [19]. Recent advances in understanding plant heat tolerance have highlighted various TFs and functional genes that regulate the physiological and molecular mechanisms activated under heat stress [20,21]. The advent of RNA sequencing has facilitated the identification of numerous genes involved in the response to extreme high temperature in fire-resistant tree species, such as the heat stress response genes observed in *Michelia macclurei* Dandy following fire stimulation [22]. Despite these advances, comprehensive insights into the mechanism underlying the response to extreme heat induced by high temperature remain limited.

This study aims to identify key genes involved in the response and recovery processes of *S. superba* under extreme heat stress, using transcriptome analysis of leaves subjected to heat stress and subsequently recover. The findings will contribute to understanding the molecular regulatory networks that underpin high temperature tolerance mechanisms in *S. superba*, providing a basis for developing new fire prevention strategies to enhance ecosystem resilience to extreme temperature conditions.

## 2. Materials and Methods

### 2.1. Plant Materials and Treatments

The seedlings of *S. superba* were planted in Nanjing Botanical Garden Mem. Sun Yat-Sen (Nanjing, China). Given the potential for this species to encounter high temperatures due to fire, we subjected the seedlings to heat stress treatment using the flame of an alcohol lamp, following the method outlined in our previous publication [22]. To capture the dynamic changes in gene expression, fresh leaves were collected from the periphery of the flame after heat treatments of 10 s, 30 s, and 1 min. Untreated leaves served as the control group (CK), and leaves collected after a 24 h recovery period following the 1 min heat treatment were designated as the recovery group. The experimental groups were labeled CK, S1, S2, S3, or S4, corresponding to the control group, 10 s, 30 s, 1 min treatment groups, and the recovery group, respectively. All collected leaves were immediately frozen in liquid nitrogen and stored at −80 °C until RNA extraction.

### 2.2. RNA Extraction, Library Construction, and Transcriptome Sequencing

The total RNA was extracted using the Plant RNA Kit (R6827, Omega Bio-Tek, Norcross, GA, USA). RNA quality was assessed with a NanoDrop One Spectrophotometer (NanoDrop Technologies, Wilmington, DE, USA) and a Qubit 3.0 Fluorometer (Life Technologies, Carlsbad, CA, USA). The qualified RNA was used for cDNA library preparation with the MGIEasy RNA Library Prep Kit for BGI^®^. First, mRNA was enriched by oligo (dT) magnetic beads and then the mRNA fragment was obtained by adding a fragmentation. The fragmented mRNA was used to synthesize first-strand and second-strand cDNA by PCR. The double-stranded cDNA then underwent end repair, adding an A tail to the 3′ end, sequencing adapters, purification, PCR amplification, and product circularization to construct the library. The fragment size and concentration of the library were detected using an Agilent 2100 Bioanalyzer, and the high-throughput platform of DNBSEQ-T7 was used for sequencing.

### 2.3. Transcriptome Assembly and Unigene Annotation

To ensure the reliability of the analysis, the raw sequencing data was processed by filtering out the low-quality reads, including those with more than five N bases, low-quality base numbers reaching 50% of reads, adapter-contaminated reads, and repetitive sequences caused by PCR amplification. The quality control of filtered data was performed using FastQC (version 0.11.9, default parameters). The clean reads were used for transcript assembly by Trinity (version 2.11.0, parameter –min_kmer_cov 2) [23]. The cd-hit (version v4.8.1, parameters -c 0.85, -aS 0.85) was then used for transcripts clustering to obtain unigenes. BUSCO (Benchmarking Universal Single-Copy Orthologs) was used to assess the unigene integrity [24]. TransDecoder software (version 5.5.0 parameters -m 50, –single_best_only) was then used for coding region prediction of unigenes (Haas et al. 2016). Seven databases were used for unigene annotation through sequence and motif similarity searches, which were Nr, Pfam [25], Uniprot [26], KEGG [27], GO (Gene Ontology) [28], KOG/COG [29], PATHWAY [30]. Transcription factors were identified by the plant database PlantTFDB (version 5.0) [31].

### 2.4. Gene Expression Quantification and Differentially Expressed Genes Analysis

The number of reads mapped to each unigene for each sample were obtained using RSEM [32] and converted to FPKM (Fragments Per Kilobase per Million bases). FPKM represents the average number of fragments per kilobase length of a gene per million fragments and is the most commonly used method for estimating gene expression levels. Paired-end reads from the same fragment were counted as one fragment, and the expression quantification of the unigene was then calculated. To detect the repeatability between samples, the Pearson correlation coefficient was calculated based on the FPKM expression level of unigenes in each pair of samples. Differential expression analysis was performed using DESeq2 software (version: 1.26.0), with a screening threshold of *p* value < 0.05 and |log2FoldChange| > 1 [33]. The differentially expressed unigenes were subjected to volcano plot analysis, cluster analysis, and enrichment analysis. Enrichment analysis included Gene Ontology and KEGG Pathway using the clusterProfiler software (version: 3.14.3) [34]. The heatmaps of the DEGs were generated by TBtools-II [35].

### 2.5. Quantitative Real-Time PCR (qRT-PCR) Validation

To validate the transcriptome data, 16 DEGs involved in heat stress were randomly selected for a quantitative real-time PCR (qRT-PCR) experiment. The experiment included primer design, cDNA synthesis, qRT-PCR, and data analysis. The gene primers (Table S1) were designed by Primer3 Release 2.3.4 and synthesized by General Biosystems Co., Ltd. (Anhui, China). According to the previous study of reference genes in *S. superba* [36], *SsuACT* (actin) was chosen as the reference gene. First-strand cDNA was synthesized from 0.5 μg isolated RNA using the PrimeScriptTM RT Reagent Kit with gDNA Eraser (TaKaRa, Dalian, China). SYBR^®^ Premix Ex Taq™ II (Tli RNaseH Plus) (TaKaRa, RR820A, Dalian, China) was used for qRT-PCR according to the protocol. The LightCycler 480 (Roche Molecular Biochemicals, Mannheim, Germany) was used for qRT-PCR experiment. Three biological and technical replicates were set for the quantification of each unigene. The relative expression level was calculated using the 2^−ΔΔCT^ method [37]. The standard errors of deviation were calculated using the STDEVA function in Excel.

## 3. Results

### 3.1. Statistics of the Transcriptome Sequencing in S. superba

A total of 15 transcriptome libraries of *S. superba* were constructed in this study. In each library, more than six Gb clean bases and approximately 43 million clean reads were obtained (Table S2). The Q20 rate (the percentage of base with Phred value ≥20) was more than 97.979% and Q30 was more than 94.226%, with the GC content 44.214–45.374% (Table S2). After assembly, a total of 336,090 transcripts and 160,449 unigenes (Table S3) were obtained, with N50 lengths of 1682 bp and 1357 bp, respectively (Figure S1A,B). BUSCO (Benchmarking Universal Single-Copy Orthologs) analysis showed that the complete BUSCOs of transcripts and unigenes reached 97.4% and 82.6% respectively (Figure S1C,D).

### 3.2. Unigene Annotation and Transcription Factors (TFs) Identification

**Figure 1:**
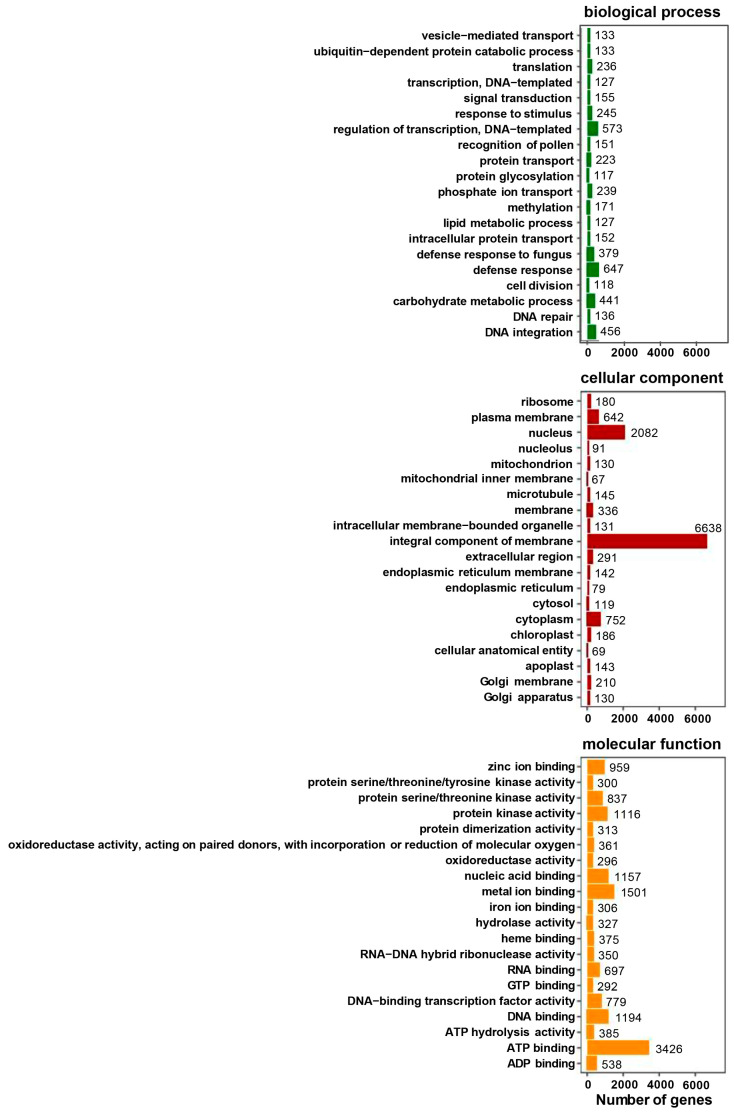
Gene ontology (GO) classification of *S. superba*.

Unigenes were annotated across seven major databases, with 34,308 (21.38% of all) unigenes annotated in at least one database. The largest amount of unigenes were annotated in the Nr database (21.19%), followed by Uniprot (21.00%), GO (15.02%), KEGG (12.61%), Pfam (10.37%), and KOG (0.12%) (Table 1). GO annotation consisted of three categories: biological process (BP), cellular component (CC), and molecular function (MF), and the top 20 terms of each category are shown in Figure 1. Most unigenes (6638) were annotated in terms of the integral component of membrane that belongs to the cellular component. Three thousand four hundred twenty-six unigens were annotated in ATP binding term (molecular function category). In the biological process, the number of annotated unigenes was much smaller than in the other categories, where the most unigenes were annotated in terms of defense response (Figure 1).

**Figure 2:**
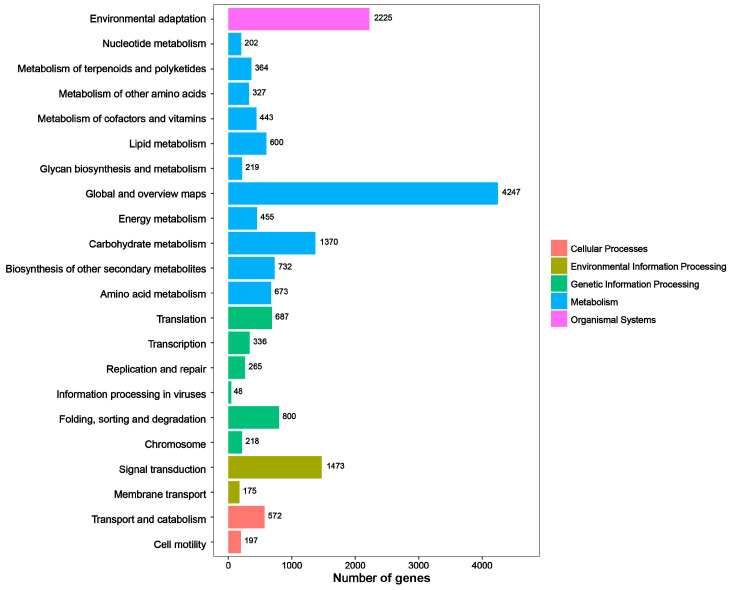
Kyoto Encyclopedia of Genes and Genomes (KEGG) classification of *S. superba*.

**Figure 3:**
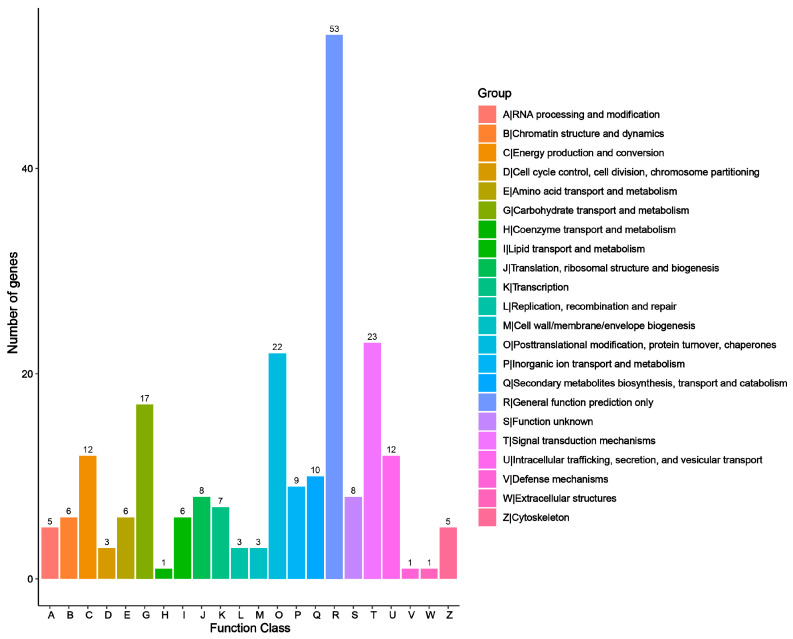
EuKaryotic Ortholog Groups (KOG) classification of *S. superba*.

In KEGG pathway annotation, unigenes were classified into 22 terms across five categories (Figure 2). The organismal systems category contained only one term related to environmental adaptation, encompassing 2225 annotated unigenes. The metabolism category contained 11 terms and had the highest number of unigenes, with 4247 annotated in terms of global and overview maps and 1370 in carbohydrate metabolism. The genetic information processing category consisted of five terms, with 800 unigenes annotated in terms of folding, sorting, and degradation. In the category of environmental information processing, 1473 unigenes were annotated for signal transduction and 175 for membrane transport. In terms of transport and catabolism, 572 unigenes were annotated. In the KOG database, orthologous unigenes were classified into 22 groups. The top groups, based on the number of annotated unigenes, were general function prediction only, signal transduction mechanisms, and posttranslational modification, protein turnover, chaperones (Figure 3). Species homology comparison in the Nr database showed that 73.31% of *S. superba* unigenes were aligned to *Camellia sinensis*, followed by *C. sinensis* var. *sinensis*, accounting for 7.83% (Figure S2A). A total of 1504 TFs were identified and classified into 52 families (Table S4), with the top 20 families shown in Figure S2B. The majority of TFs were families of MYB and MYB-related AP2 and ERF, accounting for 10.509%, 10.008%, 9.59%, and 9.34% respectively (Figure S2B).

### 3.3. Unigenes Quantification and Differentially Expressed Genes (DEGs) Analysis

**Figure 4:**
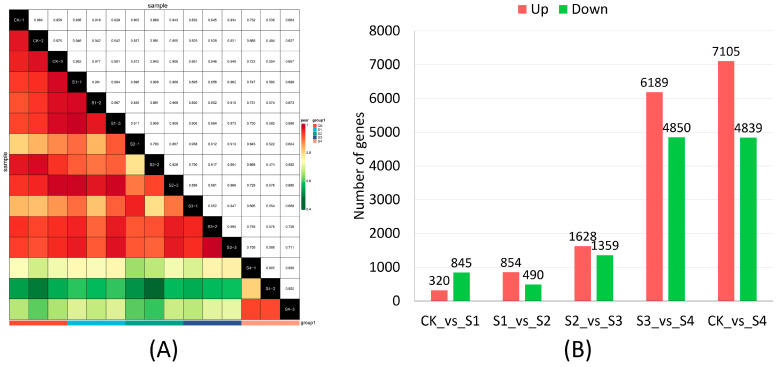
Pearson analysis of sample correlation (**A**) and the number of differentially expressed genes (DEGs) in different comparison groups (**B**). (**A**) The horizontal and vertical axes represent each sample respectively. The color depth represents the size of the correlation coefficient between the two samples. The closer the color is to red, the better the correlation between the two samples. The closer the correlation coefficient is to 1, the higher the similarity of the expression patterns between the samples. (**B**) The horizontal axis represents different comparisons, the vertical axis represents the number of unigenes, and red and green represent up- and down-regulated unigenes, respectively.

The results showed that the correlation coefficient in major sample comparisons was above 0.9 (Figure 4A). Using the criteria for identifying differentially expressed genes (DEGs), the number of DEGs was identified between the two groups (Figure 4B). Comparisons between the groups revealed that, relative to the CK group, most unigenes were up- and down-regulated in the S4 group. In the comparison between S3 and S4, the number of DEGs was similar to that in the comparison between CK and S4, but significantly higher than in the comparison between CK and S1, S1 and S2, and S2 and S3 (Figure 4B). Detailed information on DEGs is provided in Table S5.

**Figure 5:**
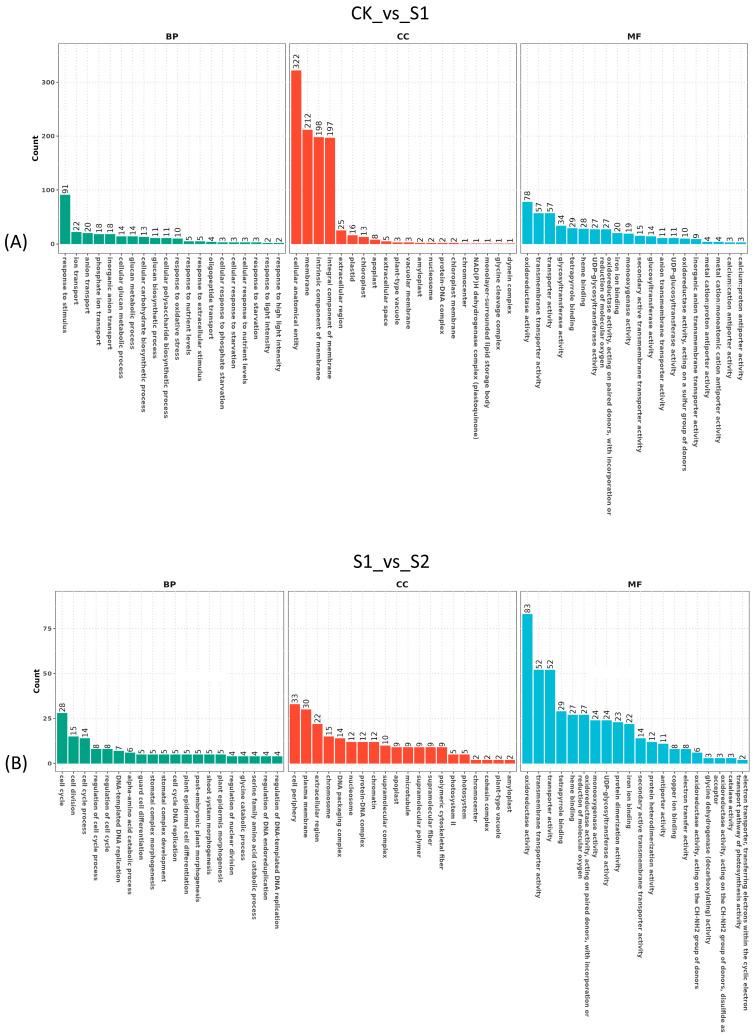
The GO enrichment of differentially expressed genes in *S. superba*. (**A**,**B**) represent the comparisons of CK_vs_S1 and S1_vs_S2.

**Figure 6:**
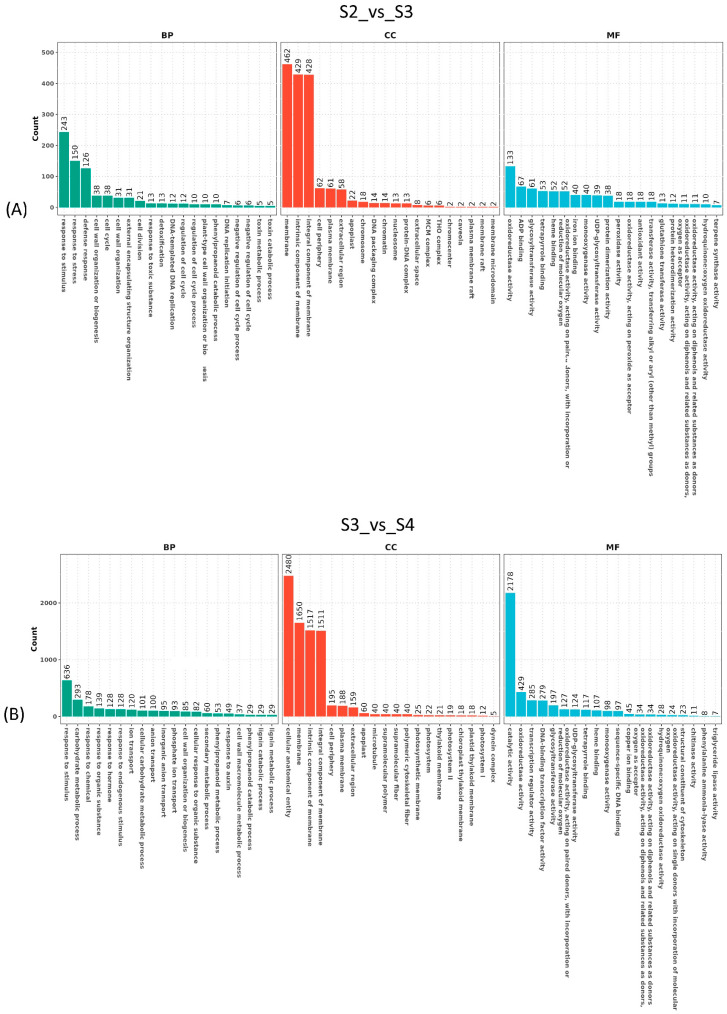
The GO enrichment of differentially expressed genes in *S. superba* (**A**,**B**) represent the comparisons of S2_vs_S3 and S3_vs_S4.

To investigate the specific functional categories of DEGs, GO and KEGG pathway enrichment were conducted. In the GO enrichment analysis, the top 20 terms in each category are shown in Figure 5 and Figure 6. Compared to the CK group, when the plant suffered a short high-temperature stimulation, the largest amount of unigenes changed in the cellular component category, such as cellular anatomical entity, membrane and intrinsic component of membrane (Figure 5A). Compared to short heat stress (10 s), when the leaves suffered 30 s stress, DEGs were significantly enriched in term of oxidoreductase activity (Figure 5B). As the heat stress duration increased to 1 min, a growing number of DEGs were enriched in terms of membrane, intrinsic component of membrane, and integral component of membrane (Figure 6A). During the recovery process, the dominant DEGs were enriched in the cellular component and molecular function, including the terms of cellular anatomical entity, catalytic activity, and membrane (Figure 6B).

**Figure 7:**
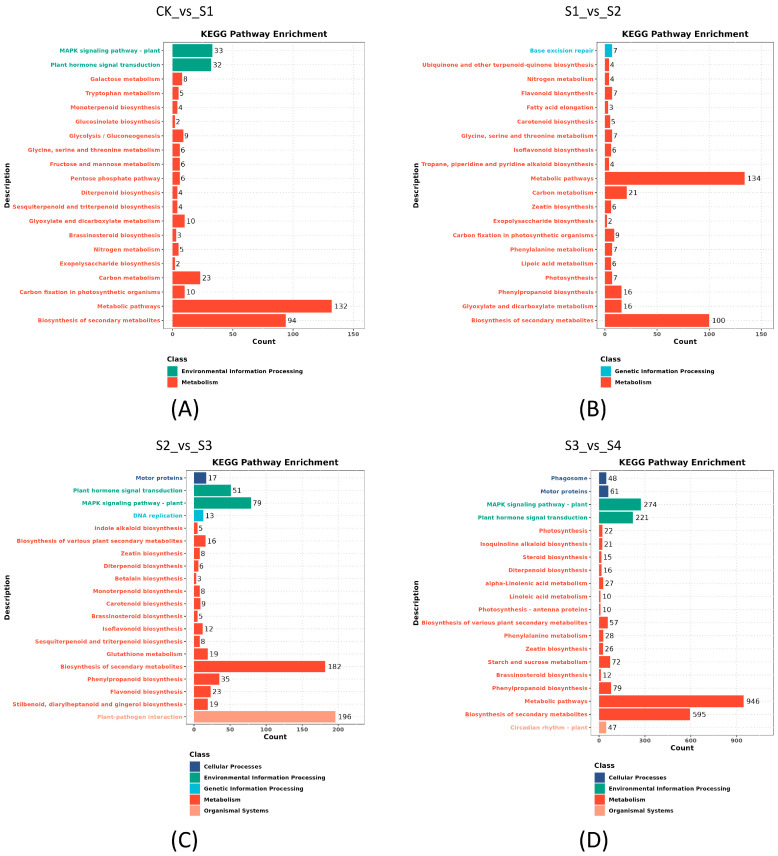
The KEGG enrichment of differentially expressed genes in *S. superba*. (**A**–**D**) represents the different comparisons between samples.

KEGG enrichment revealed the top 20 terms across four comparisons (Figure 7). The metabolism class was the predominant term that enriched in four comparisons. Within this class, the term of metabolism pathways was enriched in three comparisons and had the most DEGs, followed by the term of biosynthesis of other secondary metabolites (Figure 7A,B,D). The second major class was the environmental information processing, which contained two terms of MAPK signaling pathway and plant hormone signal transduction. These terms were enriched in three of the comparisons, excluding S1_vs_S2 (Figure 7A,C,D). The cellular processes class contained two terms of motor proteins and phagosome, with both enriched in the comparison of S3_vs_S4, while only the motor proteins term was enriched in comparison of S2_vs_S3. The plant-pathogen interaction term, part of the organismal systems class, was only enriched in the comparison of S2_vs_S3, and had 196 DEGs, representing the most unigenes. The term circadian rhythm was enriched in the comparison of S3_vs_S4 (Figure 7).

### 3.4. Differentially Expressed Genes Response to Strong Heat Stress in S. superba

**Figure 8:**
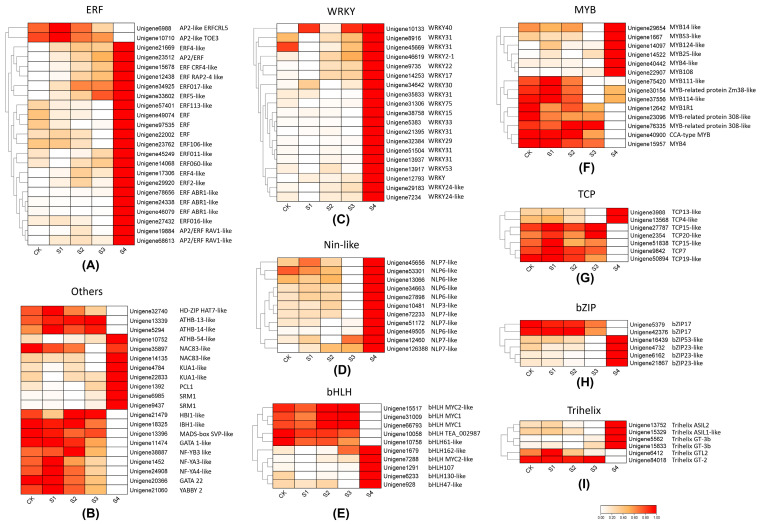
Heatmap of the differentially expressed transcription factors involved in heat response and recovery in *S. superba*. Red means up-regulation, and white means down-regulation. (**A**), (**C**–**I**) represent different TF families, and (**B**) contains several TFs that changed expression levels in response to heat stress.

To explore the key genes involved in the response and recovery process of high heat stimulation by fire in *S. superba*, differentially expressed key TFs and genes were identified across five sample groups. Eight main classes of TFs were summarized that expressed differentially in response to high temperature. Among these, ethylene-responsive transcription factor was predominant, with most genes up-regulated in the S4 group. These genes exhibited various expression patterns in the five sample groups. For example, some genes showed a gradual up-regulating trend from CK to S4, including *unigene23512* (AP2/ERF), *unigene15678* (ERF CRF4-like), *unigene12438* (ERF RAP2-4 like), *unigene34925* (ERF017-like), and *unigene33602* (ERF5-like). Conversely, ERF113-like (*unigene 57401*) and two other ERFs (*unigene49074* and *unigene97535*) had decreased expression with the increase of high-temperature stimulation time, but had significantly higher expression levels in plants that recovered for 24 h compared to the CK group. Three ERF ABR1-like unigenes did not show significant expression changes after hyperthermia stimulation compared with the CK group but were significantly up-regulated after 24 h of recovery. Two AP2-like unigenes (*unigenes6988* and *unigene10710*) showed the highest expression level in the S1 group and lowest expression in recovery plants (Figure 8A). After high-temperature stimulation recovery, WRKY family unigenes were up-regulated compared to the CK and S1-S3 groups (Figure 8C). The Nin-like family showed a similar expression pattern to the WRKY family, with the highest expression in recovery plants (Figure 8D). Unlike the WRKY and Nin-like families, the bHLH, MYB, TCP, bZIP and Trihelix families exhibited diverse expression patterns in CK and S4 groups (Figure 8E–I). Beyond the eight main TFs, some other TFs also showed high or low expression in recovery plants. For example, the NAC83-like, KUA1-like and SRM1 families were up-regulated in S4, but the genes of HD-ZIP, ATHB-13, ATHB-14, HBI1-like, NF-YB3, NF-YA3, NF-YA4, and YABBY2 were down-regulated in S4 (Figure 8B).

**Figure 9:**
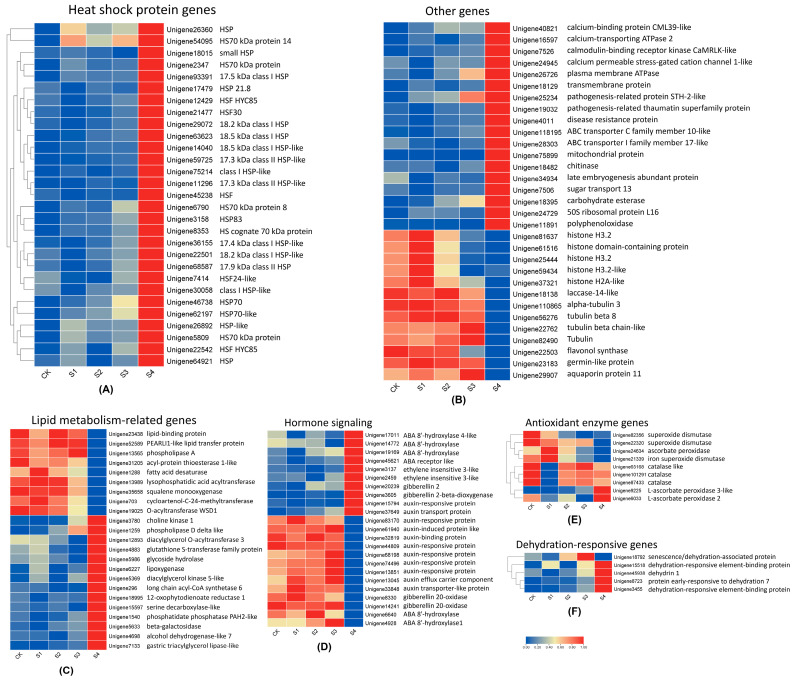
Heatmap of the differentially expressed functional genes related to heat response and recovery in *S. superba*. (**A**) Heat shock protein genes; (**B**) Other genes; (**C**) Lipid metabolism-related genes; (**D**) Hormone signaling; (**E**) Antioxidant enzyme genes; (**F**) Dehydration-responsive genes. Red means up-regulation, and blue means down-regulation.

In addition to transcription factors, many important functional genes are involved in the biological processes of heat response and recovery after stimulation. These include genes related to heat shock protein genes, antioxidant enzyme genes, dehydration-responsive genes, and lipid metabolism-related genes. The heat shock protein genes were significantly up-regulated in S4, including 24 HSPs and five HSFs (Figure 9A). Dehydration-responsive genes also exhibited their highest expression in S4, except for *unigene18792* (senescence/dehydration-associated protein), which reached the highest expression level and decreased after 24 h of recovery. Regarding antioxidant enzyme genes, those related to superoxide dismutase, ascorbate peroxidase, and iron superoxide dismutase showed decreased expression after high-temperature stimulation and recovery. However, L-ascorbate peroxidase genes were up-regulated. Catalase genes were significantly down-regulated after a 10 s fire stimulation but later up-regulated to a level close to the CK group (Figure 9E). Lipid metabolism, a complex biological process, involves many genes that are either up- or down-regulated in response to high temperature. For example, the lipid-binding protein gene and lipid transfer protein genes decreased expression in S4, while genes related to choline kinase, glycoside hydrolase, and lipoxygenase were up-regulated (Figure 9C). In plant hormone signal transduction, ethylene insensitive genes were up-regulated after heat stress and recovery. Conversely, many genes related to auxin-responsive protein, auxin-induced protein, and auxin-binding protein were down-regulated in S4. Genes of gibberellin 2-oxidase had the highest expression in S4, whereas gibberellin 20-oxidase genes showed decreased expression in S4. Abscisic acid (ABA) 8′-hydroxylase showed different expression patterns in S4 (Figure 9D).

### 3.5. qRT-PCR Validation of DEGs

**Figure 10:**
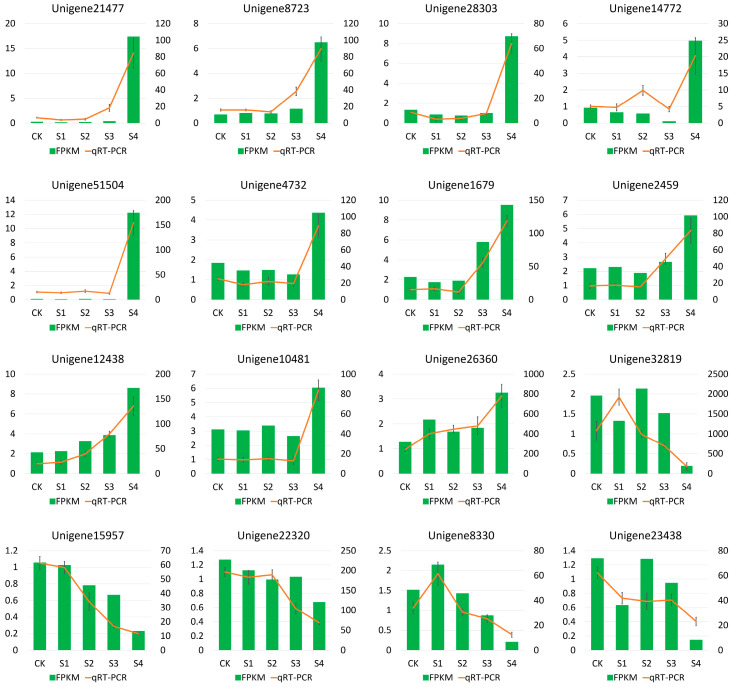
qRT-PCR validation of 16 DEGs involved in heat stress response in *S. superba*. The x-axis represents the five sample groups, and the y-axis shows the FPKM by RNA-Seq and the relative quantitative expression level by qRT-PCR for each unigene.

To validate the accuracy of RNA-Seq, 16 DEGs related to heat stress response were randomly selected for qRT-PCR. The results showed a high degree of consistency between RNA-Seq and qRT-PCR (Figure 10). For instance, genes such as HSF30 (*Unigene21477*), protein early-responsive to dehydration (*Unigene8723*), ABC transporter (*Unigene28303*), ABA 8′-hydroxylase (*Unigene14772*) and WRKY31 (*Unigene51504*) exhibited significant up-regulation in the group that had undergone 24 h of recovery after heat stress. On the contrary, genes related to auxin-binding protein (*Unigene32819*), MYB4 (*Unigene15957*), and gibberellin 20-oxidase (*Unigene8330*) had the lowest expression levels in the recovery group (Figure 10).

## 4. Discussion

Plant responses and recovery to high heat stress involve many genes and biological processes, forming complex transcriptional regulatory networks [20,38]. Transcriptome analysis in many plants, such as switchgrass [39], potato [40], and rice [41] has identified many genes related to high heat stress. *S. superba*, an important fire-resistant tree species, has previously been studied for its leaves’ physiological and biochemical response to smoke by simulating forest fire [18]. However, the molecular mechanisms underlying its response to high temperature remain underexplored. In this study, transcriptomic analysis of *S. superba* under fire-induced high temperature stress and subsequent recovery provides significant insights into the genetic and molecular mechanisms that enable this species to withstand and recover from such extreme temperature conditions.

GO and KEGG functional annotation of unigenes revealed the diverse biological processes, molecular functions, and cellular components involved in stress response and recovery. Differentially expressed genes exhibited significant changes between the control (CK) and recovery groups by heat stress in *S. superba* leaves. This suggests a sophisticated regulatory network that coordinates the plant’s response to heat stress and subsequent recovery. HSPs, a main group of molecular chaperones, are highly conserved in plants and are essential for maintaining protein folding, stability, and cellular homeostasis under heat stress conditions [42,43,44,45]. The identified HSPs encompass various families from many species, such as 21 HSFs encoding genes in Arabidopsis [46], 56 in wheat [47], etc. These HSPs perform diverse biological functions to prevent cellular toxicity under prolonged heat exposure. For example, overexpression of Arabidopsis HSFs can enhance tolerance to heat and other stresses [48]. Additionally, 25 *ZmHsf* and 22 *ZmHsp*70 genes were identified as having potential effects on heat stress response [49], and 141 Hsf-like and Hsp-like genes were identified in eggplant, with most increasing after heat stress [50]. In this study, upon exposure to fire and 24 h of recovery, 24 HSPs and five HSFs exhibited pronounced up-regulation after recovery, suggesting their pivotal role in protecting cellular proteins and reflecting the immediate adaptive response to mitigate the detrimental effects of thermal stress on cellular components.

The activation of oxidative metabolism has been shown to overlap with the transcription of HSFs and HSPs during heat stress, indicating its integral role in the heat response and enhanced heat tolerance of plants [51]. Under high temperature stimulation, some specific antioxidant enzyme genes will be activated. Enzymes such as superoxide dismutase (SOD), peroxidase (POD), ascorbate peroxidase (APX), and catalase (CAT) typically respond more significantly. For instance, these enzymes were found to be increased in the heat-tolerant genotypes of *Cucurbita moschata* (Duchesne ex Lam.) Duchesne ex Poir. [52]. Similarly, high-temperature treatment in mulberry cultivars led to elevated antioxidant enzyme activities, including SOD, CAT, POD, APX, and glutathione reductase (GR) [53]. Over-expressed *CgHSP70* plants showed higher POD activity and proline content in *Chrysanthemum* [54], suggesting that these enzymes could help clear reactive oxygen in plants and reduce oxidative damage after heat stress. In *S. superba*, CAT genes, which are essential for hydrogen peroxide breakdown, were initially down-regulated after heat stress, but increased in the longer heat stimulation and recovery groups. The genes of SOD and ascorbate peroxidase were down-regulated after extreme heat stress and decreased to a much lower expression than the CK group, while L-ascorbate peroxidase genes showed high expression in the recovery group. This indicates that CAT and L-ascorbate peroxidase genes facilitate the clearance of reactive oxygen after heat stress and reach a balance with SOD and POD to manage oxidative stress. Together, these enzymes are regulated to protect cells from ROS damage and facilitate recovery after exposure to extremely high temperatures.

Reactive oxygen species induced by heat stress can cause damage to lipids and proteins in plants, which affects the stability of plant cell membranes. This triggers changes in the expression of some related genes in lipid metabolism pathways to maintain membrane integrity and function [55,56]. This study identified a series of lipid metabolism-related genes showing a complex response to high temperature. Genes related to lipid-binding protein, lipid transfer protein, phospholipase A, and fatty acid desaturase decreased after long heat stress and reached their lowest expression after 24 h of recovery. However, genes for choline kinase, glycoside hydrolase, and lipoxygenase were up-regulated after recovery from heat stress. In grass species, heat stress enhances the accumulation of phospholipids and glycolipids [57], suggesting that the ability of lipid binding and transfer decrease after heat stress. The up-regulated genes may participate in the biosynthesis of phospholipids and glycolipids during lipid metabolism, promoting tolerance and recovery potential from extreme heat stress in *S. superba*. During the recovery phase, gene expression towards the restoration of cellular functions is disrupted by heat stress. Genes associated with photosynthesis and carbon metabolism, such as calcium-binding related genes, transmembrane protein, and mitochondrial protein genes showed gradual recovery. This indicates the plant’s ability to rebuild energy reserves and metabolic pathways essential for growth and development. This adaptive response likely supports the regeneration of damaged tissues and enhances overall resilience following high temperatures.

In addition, it has been proven that all major plant hormones play a critical role in response to heat stress, including auxin, abscisic acid (ABA), gibberellins, and ethylene [58]. ABA is a key hormone involved in abiotic stress responses. In the context of heat stress, the ABA regulatory network includes several HSFs, Dehydration-Responsive Element Binding Protein 2A (DREB2A), bZIP, MYB, and NAC proteins. DREB2A induces the expression of *HSFA3* under high temperatures, thereby activating many stress-related genes [14,59]. In *S. superba*, dehydration-responsive genes closely related to ABA were significantly up-regulated after heat stress, and the expression patterns of ABA 8′-hydroxylase, which is involved in ABA catabolism, varied during the recovery phase (S4). Differential expression of ABA-related genes indicates a complex regulatory mechanism. Increased ABA-related gene expression after heat stress can enhance stress tolerance by promoting stomatal closure and reducing water loss, while reduced levels during recovery can facilitate growth resumption. This dynamic regulation of ABA metabolism has been observed in other plants, such as *Triticum aestivum* L., where ABA levels are closely regulated during drought and heat stress to optimize survival and growth [60]. Gibberellin-related genes also showed different expression patterns; gibberellin-2-oxidase genes had the highest expression in S4, while gibberellin 20-oxidase genes decreased in expression. In plants, high temperature also commonly suppresses GA and IAA biosynthesis [61,62]. Most of the auxin-response and auxin-binding genes were down-regulated in S4, which may have a common function with decreased ABA genes to the response of high temperature in *S. superba*.

In plants, various transcription factors and functional genes play crucial roles in responding to environmental stresses. In this study, a total of 1504 TFs were identified and classified into 52 families, with ERF, WRKY and MYB being the most prominent. In other plants, families such as AP2/ERF, MYB, WRKY, and bZIP have been shown to play significant roles in heat stress responses, as seen in rice [63] and fire-resistant tree species *M. macclurei* [22]. Ethylene responsive factors (ERFs) are known to mediate plant responses to abiotic stresses by regulating genes involved in ethylene signaling, stress tolerance, and recovery processes [64,65]. In *S. superba*, most ERFs exhibited a gradually increasing pattern from CK to S4, peaking after 24 h of recovery, accompanied by increasing expression of ethylene-related genes. This indicates their involvement in stress response and recovery. The WRKY family showed up-regulation in the recovery phase compared to CK and S1-S3 groups, suggesting their role in restoring normal cellular functions post-stress. Nin-like (NLP) TFs exhibited a similar expression pattern to the WRKY family, with the highest expression in recovery plants but low expression in long heat stress of S3 group, indicating a potential collaborative role in stress recovery. In *Capsicum annuum* (L.), the expression of *CaNLP1* increased after various abiotic stress [66], which is consistent with the expression in *S. superba*’s response to heat stress. Other families including MYB, bHLH, bZIP, TCP, Trihelix, NAC and NF-Y exhibited diverse expression patterns in CK and S4 groups. These TFs control diverse biological processes in plants, such as development and abiotic stress responses [67,68]. For example, the expression of TCP gene changes in response to abiotic stresses including heat, drought, and salt in ginger [69]. Under heat stress treatment, *PgMYB2*, *PgMYB9*, *PgMYB88* and *PgMYB151* are differentially expressed in *Pennisetum glaucum* (L.) R. Br. [70]. The diversity of these TFs in *S. superba* reflects their varied roles in different stages of heat response and recovery, potentially regulating a broad spectrum of stress-responsive genes.

## 5. Conclusions

In this study, 15 transcriptomes of *S. superba* subjected to heat stress and 24 h of recovery were assembled, revealing the primary molecular and biological pathways of response to high temperature in this fire-resistant tree species. Analysis of differentially expressed genes identified key candidate genes and transcription factors involved in the heat stress response in *S. superba*. Ethylene-responsive factors, WRKY, MYB, bHLH, and Nin-like families play a central role in regulating the networks of heat stress response and adaption, while other TF families contribute to different aspects of stress. Functional genes related to heat shock proteins/factors, lipid metabolism, antioxidant enzymes, dehydration response, and hormone signal transduction factors are crucial for maintaining cellular homeostasis during and after exposure to high temperatures. Changes in several hormonal-related genes indicate more complexity and stress response mechanisms. These findings provide comprehensive insights into the molecular mechanisms governing the response and recovery of *S. superba* to high temperatures. The identified key genes not only enhance our understanding of plant defense and resilience strategies but also offer potential genes for future genetic and breeding studies to enhance heat tolerance in economically important plant species facing increasing climate variability and extreme events.

## Figures and Tables

**Table 1:** Statistics of unigene annotation in *Schima superba*.

Item.	Count	Percentage
Nr	34,006	21.19%
Uniprot	33,691	21.00%
GO	24,105	15.02%
KEGG	20,238	12.61%
Pfam	16,631	10.37%
Pathway	10,068	6.27%
KOG	195	0.12%
Annotation	34,308	21.38%
All	160,449	100.00%

## Data Availability

All data are available on reasonable request to the corresponding authors.

## Supplementary Materials

The following supporting information can be downloaded at: https://www.mdpi.com/article/10.3390/genes15081108/s1, Figure S1: Length distribution and quality assessment of transcripts and unigenes; Figure S2: Species distribution and transcription factor family proportion of unigenes in Nr annotation; Table S1: Primers used for qRT-PCR analysis; Table S2: Statistic of the transcriptome in *S. superba*; Table S3: All assembled unigenes in *S. superba*; Table S4: Identified transcription factor in *S. superba*; Table S5: Differentially expressed unigenes in different comparisons in *S. superba*.

## References

[1] Rocca M.E., Brown P.M., MacDonald L.H., Carrico C.M. (2014). Climate change impacts on fire regimes and key ecosystem services in Rocky Mountain forests. For. Ecol. Manag..

[2] Singh S. (2022). Forest fire emissions: A contribution to global climate change. Front. For. Glob. Chang..

[3] Noss R.F., Franklin J.F., Baker W.L., Schoennagel T., Moyle P.B. (2006). Ecology and management of fire-prone forests of the western United States. Society for Conservation Biology Scientific Panel or Fire in Western US Forests.

[4] Zahed M., Bączek-Kwinta R. (2023). The impact of post-fire smoke on plant communities: A global approach. Plants.

[5] Midgley J.J., Bond W.J. (2013). Plant adaptations to fire: An evolutionary perspective. Fire Phenomena and the Earth System: An Interdisciplinary Guide to Fire Science.

[6] Odhiambo B.O. (2015). The Effect of Fire Damage on the Growth and Survival Mechanisms of Selected Native and Commercial Trees in South Africa. Doctoral Dissertation.

[7] Rosell J.A. (2019). Bark in woody plants: Understanding the diversity of a multifunctional structure. Integr. Comp. Biol..

[8] Kregel K.C. (2002). Invited review: Heat shock proteins: Modifying factors in physiological stress responses and acquired thermotolerance. J. Appl. Physiol..

[9] Archana P., Aleena J., Pragna P., Vidya M., Niyas A., Bagath M., Krishnan G., Manimaran A., Beena V., Kurien E. (2017). Role of heat shock proteins in livestock adaptation to heat stress. J. Dairy Vet. Anim. Res..

[10] Bokszczanin K.L., Consortium S.P.T.I.T.N., Fragkostefanakis S. (2013). Perspectives on deciphering mechanisms underlying plant heat stress response and thermotolerance. Front. Plant Sci..

[11] Fragkostefanakis S., Roeth S., Schleiff E., SCHARF K.D. (2015). Prospects of engineering thermotolerance in crops through modulation of heat stress transcription factor and heat shock protein networks. Plant Cell Environ..

[12] Liu G.T., Wang J.F., Cramer G., Dai Z.W., Duan W., Xu H.G., Wu B.H., Fan P.G., Wang L.J., Li S.H. (2012). Transcriptomic analysis of grape (*Vitis vinifera* L.) leaves during and after recovery from heat stress. BMC Plant Biol..

[13] Liang M.H., Jiang J.G., Wang L., Zhu J. (2020). Transcriptomic insights into the heat stress response of *Dunaliella bardawil*. Enzyme Microb. Technol..

[14] Ohama N., Sato H., Shinozaki K., Yamaguchi-Shinozaki K. (2017). Transcriptional regulatory network of plant heat stress response. Trends Plant Sci..

[15] Haider S., Raza A., Iqbal J., Shaukat M., Mahmood T. (2022). Analyzing the regulatory role of heat shock transcription factors in plant heat stress tolerance: A brief appraisal. Mol. Biol. Rep..

[16] Yang H., Zhang R., Song P., Zhou Z. (2017). The floral biology, breeding system and pollination efficiency of *Schima superba* Gardn. et Champ. (Theaceae). Forests.

[17] Zhang R., Yang H., Zhou Z., Shen B., Xiao J., Wang B. (2019). A high-density genetic map of Schima superba based on its chromosomal characteristics. BMC Plant Biol..

[18] Zheng W., Ma Y., Tigabu M., Yi Z., Guo Y., Lin H., Huang Z., Guo F. (2022). Capture of fire smoke particles by leaves of *Cunninghamia lanceolata* and *Schima superba*, and importance of leaf characteristics. Sci. Total Environ..

[19] Zhou G., Zhou Y., Yu S., Bai S., Lu F. (2009). *Schima superba* as a fuelbreak: Litter combustibility of three tree species with five water content levels using a cone calorimeter. Front. For. China.

[20] Haider S., Iqbal J., Naseer S., Yaseen T., Shaukat M., Bibi H., Ahmad Y., Daud H., Abbasi N.L., Mahmood T. (2021). Molecular mechanisms of plant tolerance to heat stress: Current landscape and future perspectives. Plant Cell Rep..

[21] Manna M., Thakur T., Chirom O., Mandlik R., Deshmukh R., Salvi P. (2021). Transcription factors as key molecular target to strengthen the drought stress tolerance in plants. Physiol. Plant..

[22] Wei S., Song Z., Luo S., Zhong Y., Zhou Y., Lu R. (2023). Transcriptome analysis reveals the heat stress response genes by fire stimulation in *michelia macclurei* dandy. Forests.

[23] Grabherr M.G., Haas B.J., Yassour M., Levin J.Z., Thompson D.A., Amit I., Adiconis X., Fan L., Raychowdhury R., Zeng Q. (2011). Full-length transcriptome assembly from RNA-Seq data without a reference genome. Nat. Biotechnol..

[24] Manni M., Berkeley M.R., Seppey M., Simão F.A., Zdobnov E.M. (2021). BUSCO update: Novel and streamlined workflows along with broader and deeper phylogenetic coverage for scoring of eukaryotic, prokaryotic, and viral genomes. Mol. Biol. Evol..

[25] Finn R.D., Bateman A., Clements J., Coggill P., Eberhardt R.Y., Eddy S.R., Heger A., Hetherington K., Holm L., Mistry J. (2014). Pfam: The protein families database. Nucleic Acids Res..

[26] (2021). UniProt: The universal protein knowledgebase in 2021. Nucleic Acids Res..

[27] Kanehisa M., Goto S., Kawashima S., Okuno Y., Hattori M. (2004). The KEGG resource for deciphering the genome. Nucleic Acids Res..

[28] Ashburner M., Ball C.A., Blake J.A., Botstein D., Butler H., Cherry J.M., Davis A.P., Dolinski K., Dwight S.S., Eppig J.T. (2000). Gene ontology: Tool for the unification of biology. Nat. Genet..

[29] Tatusov R.L., Fedorova N.D., Jackson J.D., Jacobs A.R., Kiryutin B., Koonin E.V., Krylov D.M., Mazumder R., Mekhedov S.L., Nikolskaya A.N. (2003). The COG database: An updated version includes eukaryotes. BMC Bioinform..

[30] Kanehisa M., Furumichi M., Tanabe M., Sato Y., Morishima K. (2017). KEGG: New perspectives on genomes, pathways, diseases and drugs. Nucleic Acids Res..

[31] Jin J., Tian F., Yang D.C., Meng Y.Q., Kong L., Luo J., Gao G. (2016). PlantTFDB 4.0: Toward a central hub for transcription factors and regulatory interactions in plants. Nucleic Acids Res..

[32] Li B., Dewey C.N. (2011). RSEM: Accurate transcript quantification from RNA-Seq data with or without a reference genome. BMC Bioinform..

[33] Love M.I., Huber W., Anders S. (2014). Moderated estimation of fold change and dispersion for RNA-seq data with DESeq2. Genome Biol..

[34] Yu G., Wang L.G., Han Y., He Q.Y. (2012). clusterProfiler: An R package for comparing biological themes among gene clusters. Omics J. Integr. Biol..

[35] Chen C., Wu Y., Li J., Wang X., Zeng Z., Xu J., Liu Y., Feng J., Chen H., He Y. (2023). TBtools-II: A “one for all, all for one” bioinformatics platform for biological big-data mining. Mol. Plant.

[36] Yang Z., Zhang R., Zhou Z. (2021). Identification and validation of reference genes for gene expression analysis in *Schima superba*. Genes.

[37] Livak K.J., Schmittgen T.D. (2001). Analysis of relative gene expression data using real-time quantitative PCR and the 2^−ΔΔCT^ method. Methods.

[38] Kotak S., Larkindale J., Lee U., von Koskull-Döring P., Vierling E., Scharf K.D. (2007). Complexity of the heat stress response in plants. Curr. Opin. Plant Biol..

[39] Li Y.-F., Wang Y., Tang Y., Kakani V.G., Mahalingam R. (2013). Transcriptome analysis of heat stress response in switchgrass (*Panicum virgatum* L.). BMC Plant Biol..

[40] Tang R., Gupta S.K., Niu S., Li X.Q., Yang Q., Chen G., Zhu W., Haroon M. (2020). Transcriptome analysis of heat stress response genes in potato leaves. Mol. Biol. Rep..

[41] Yang Y., Zhang C., Zhu D., He H., Wei Z., Yuan Q., Li X., Gao X., Zhang B., Gao H. (2022). Identifying candidate genes and patterns of heat-stress response in rice using a genome-wide association study and transcriptome analyses. Crop J..

[42] Usman M.G., Rafii M., Ismail M., Malek M., Latif M.A., Oladosu Y. (2014). Heat shock proteins: Functions and response against heat stress in plants. Int. J. Sci. Technol. Res..

[43] Feder M.E., Hofmann G.E. (1999). Heat-shock proteins, molecular chaperones, and the stress response: Evolutionary and ecological physiology. Annu. Rev. Physiol..

[44] Tkáčová J., Angelovičová M. (2012). Heat shock proteins (HSPs): A review. Sci. Pap. Anim. Sci. Biotechnol..

[45] Jacob P., Hirt H., Bendahmane A. (2017). The heat-shock protein/chaperone network and multiple stress resistance. Plant Biotechnol. J..

[46] Scharf K.D., Berberich T., Ebersberger I., Nover L. (2012). The plant heat stress transcription factor (Hsf) family: Structure, function and evolution. Biochim. Biophys. Acta (BBA)-Gene Regul. Mech..

[47] Xue G.P., Sadat S., Drenth J., McIntyre C.L. (2014). The heat shock factor family from *Triticum aestivum* in response to heat and other major abiotic stresses and their role in regulation of heat shock protein genes. J. Exp. Bot..

[48] Qian J., Chen J., Liu Y., Yang L., Li W., Zhang L. (2014). Overexpression of *Arabidopsis HsfA1a* enhances diverse stress tolerance by promoting stress-induced *Hsp* expression. Genet. Mol. Res..

[49] Jiang L., Hu W., Qian Y., Ren Q., Zhang J. (2021). Genome-wide identification, classification and expression analysis of the *Hsf* and *Hsp70* gene families in maize. Gene.

[50] Gong C., Pang Q., Li Z., Li Z., Chen R., Sun G., Sun B. (2021). Genome-wide identification and characterization of *Hsf* and *Hsp* gene families and gene expression analysis under heat stress in eggplant (*Solanum melongema* L.). Horticulturae.

[51] Pucciariello C., Banti V., Perata P. (2012). ROS signaling as common element in low oxygen and heat stresses. Plant Physiol. Biochem..

[52] Ara N., Nakkanong K., Lv W., Yang J., Hu Z., Zhang M. (2013). Antioxidant enzymatic activities and gene expression associated with heat tolerance in the stems and roots of two cucurbit species (“*Cucurbita maxima*” and “*Cucurbita moschata*”) and their interspecific inbred line “*Maxchata*”. Int. J. Mol. Sci..

[53] Chaitanya K., Sundar D., Masilamani S., Ramachandra Reddy A. (2002). Variation in heat stress-induced antioxidant enzyme activities among three mulberry cultivars. Plant Growth Regul..

[54] Song A., Zhu X., Chen F., Gao H., Jiang J., Chen S. (2014). A chrysanthemum heat shock protein confers tolerance to abiotic stress. Int. J. Mol. Sci..

[55] Niu Y., Xiang Y. (2018). An overview of biomembrane functions in plant responses to high-temperature stress. Front. Plant Sci..

[56] Liang Y., Huang Y., Liu C., Chen K., Li M. (2023). Functions and interaction of plant lipid signalling under abiotic stresses. Plant Biol..

[57] Zhang X., Xu Y., Huang B. (2019). Lipidomic reprogramming associated with drought stress priming-enhanced heat tolerance in tall fescue (*Festuca arundinacea*). Plant Cell Environ..

[58] Ahammed G.J., Li X., Zhou J., Zhou Y.H., Yu J.Q. (2016). Role of hormones in plant adaptation to heat stress. Plant Hormones under Challenging Environmental Factors.

[59] Yoshida T., Ohama N., Nakajima J., Kidokoro S., Mizoi J., Nakashima K., Maruyama K., Kim J.M., Seki M., Todaka D. (2011). *Arabidopsis* HsfA1 transcription factors function as the main positive regulators in heat shock-responsive gene expression. Mol. Genet. Genom..

[60] Bi H., Zhao Y., Li H., Liu W. (2020). Wheat heat shock factor TaHsfA6f increases ABA levels and enhances tolerance to multiple abiotic stresses in transgenic plants. Int. J. Mol. Sci..

[61] Toh S., Imamura A., Watanabe A., Nakabayashi K., Okamoto M., Jikumaru Y., Hanada A., Aso Y., Ishiyama K., Tamura N. (2008). High temperature-induced abscisic acid biosynthesis and its role in the inhibition of gibberellin action in Arabidopsis seeds. Plant Physiol..

[62] Sakata T., Oshino T., Miura S., Tomabechi M., Tsunaga Y., Higashitani N., Miyazawa Y., Takahashi H., Watanabe M., Higashitani A. (2010). Auxins reverse plant male sterility caused by high temperatures. Proc. Natl. Acad. Sci. USA.

[63] He Y., Guan H., Li B., Zhang S., Xu Y., Yao Y., Yang X., Zha Z., Guo Y., Jiao C. (2023). Transcriptome analysis reveals the dynamic and rapid transcriptional reprogramming involved in heat stress and identification of heat response genes in rice. Int. J. Mol. Sci..

[64] Cheng M.C., Liao P.M., Kuo W.W., Lin T.P. (2013). The Arabidopsis ETHYLENE RESPONSE FACTOR1 regulates abiotic stress-responsive gene expression by binding to different cis-acting elements in response to different stress signals. Plant Physiol..

[65] Huang J., Zhao X., Bürger M., Chory J., Wang X. (2023). The role of ethylene in plant temperature stress response. Trends Plant Sci..

[66] Wu Y., Su S., Wang T., Peng G.H., He L., Long C., Li W. (2023). Identification and expression characteristics of NLP (NIN-like protein) gene family in pepper (*Capsicum annuum* L.). Mol. Biol. Rep..

[67] von Koskull-Döring P., Scharf K.-D., Nover L. (2007). The diversity of plant heat stress transcription factors. Trends Plant Sci..

[68] Song A., Li P., Jiang J., Chen S., Li H., Zeng J., Shao Y., Zhu L., Zhang Z., Chen F. (2014). Phylogenetic and transcription analysis of Chrysanthemum WRKY transcription factors. Int. J. Mol. Sci..

[69] Jiang Y., Jiang D., Xia M., Gong M., Li H., Xing H., Zhu X., Li H.L. (2023). Genome-wide identification and expression analysis of the TCP gene family related to developmental and abiotic stress in ginger. Plants.

[70] Chanwala J., Khadanga B., Jha D.K., Sandeep I.S., Dey N. (2023). MYB transcription factor family in pearl millet: Genome-wide identification, evolutionary progression and expression analysis under abiotic stress and phytohormone treatments. Plants.

