# Supplementary figures and images for: Transcriptome Analysis Identifies Key Genes Involved in Response and Recovery to High Heat Stress Induced by Fire in Schima superba
Source: Genes (Basel). 2024 Aug 22;15(8):1108. doi: 10.3390/genes15081108 (PMC11353729; doi:10.3390/genes15081108)

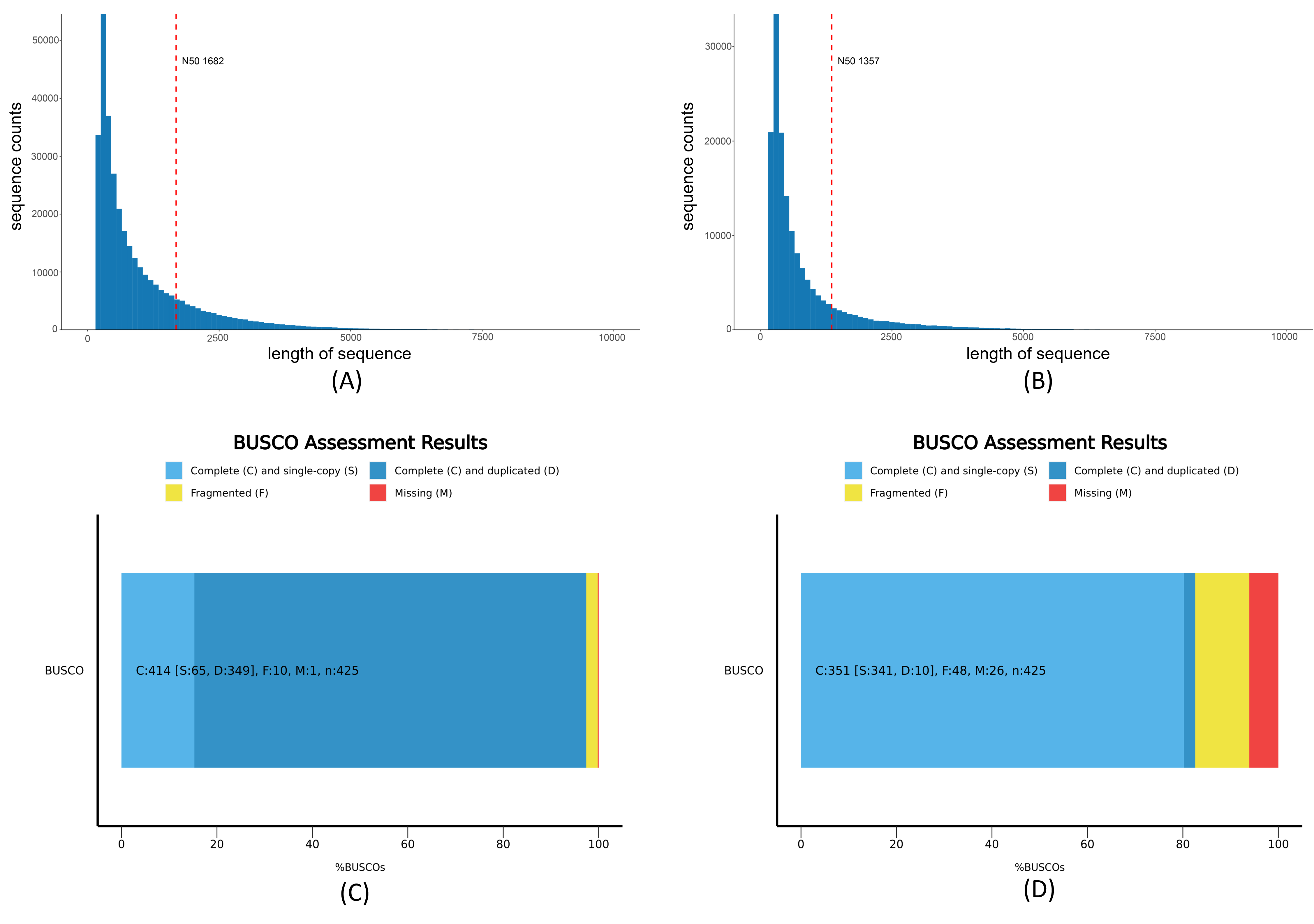

Supplement: Supplementary file 1 [file genes-15-01108-s001.zip › Figure S1 Length distribution and quality assessment of transcripts and unigenes.tif]

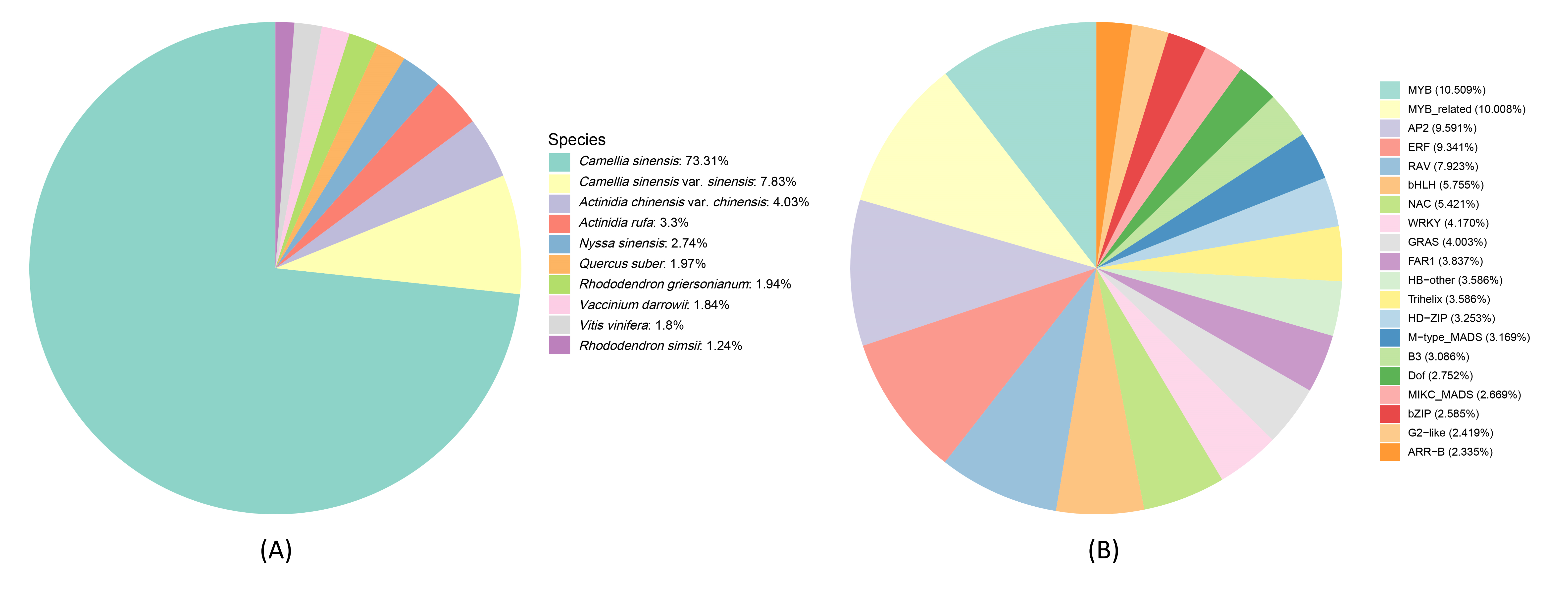

Supplement: Supplementary file 1 [file genes-15-01108-s001.zip › Figure S2 Species distribution and transcription factor family proportion of unigenes in Nr annotation.tif]
